# Syndemic effect of COVID-19 outbreak on HIV care delivery around the globe: A systematic review using narrative synthesis

**DOI:** 10.34172/hpp.2023.30

**Published:** 2023-12-16

**Authors:** Rohini Chakrabarti, Debdutta Agasty, Agniva Majumdar, Rounik Talukdar, Mihir Bhatta, Subrata Biswas, Shanta Dutta

**Affiliations:** ICMR-National Institute of Cholera and Enteric Diseases, Kolkata, India

**Keywords:** COVID-19, Pandemic, HIV, HIV care continuum, Impact, Systematic review

## Abstract

**Background:**

The burden of the COVID-19 pandemic on healthcare systems worldwide has been compromising the progress made in the fight against HIV. This paper aims to determine how the COVID-19 pandemic has impacted HIV comprehensive care service delivery globally as well as to consolidate the evidence and recommendations that may be useful in averting future crisis.

**Methods:**

This review adheres to PRISMA guidelines. PubMed, DOAJ, Science Direct and other sources like Google Scholar and citations from included studies were searched methodically to locate studies evaluating the effects of COVID on services for HIV care. The NIH and JBI quality assessment tools were used for the quality assessment of individual studies.

**Results:**

In the present review 31 eligible studies were included and the impact on HIV care cascade were summarised under six themes: Lab services, Treatment and allied services, Counselling services, Outreach services, Psycho-social impact and Implementation of sustainable strategies. The studies also presented many innovative alternatives which were lucidly highlighted in the present article.

**Conclusion:**

Current evidence depicts multiple factors are responsible for the interruption of HIV care service delivery during the pandemic, especially in low resources settings. The prospective, alternative solutions that have been used to circumvent the threat have also been addressed in this review, in addition to the negative aspects that have been observed. Transition with new innovative, sustainable care paradigms may prove to be the building blocks in removing HIV-AIDS as a public health threat.

**Registration:**

Open Science Framework (DOI: 10.17605/OSF.IO/74GHM).

## Introduction

 The first documented case of COVID-19 infection was detected in China’s Wuhan province on December 2019, and it was proclaimed a worldwide pandemic by the World Health Organization (WHO) on March of 2020.^1^ With the rapid spread across the globe, the successive waves of the pandemic have reversed the progress of various healthcare programs leaving serious short as well as long-term impacts on essential healthcare services. Many essential healthcare activities were paused temporarily and healthcare workers from different streams were diverted to COVID-19 care resulting in disruption of the services. HIV comprehensive care services being one of them were no exception. Like the general population, people living with HIV (PLHIV) were also forced to stay indoors with limited access to health facilities, loss to follow-up, and discontinuation of antiretroviral therapy.^2^

 According to the UNAIDS Global AIDS Update 2022, in 2021 HIV-AIDS took a life each minute, leading to 650 000 AIDS-related deaths worldwide. In 2021, a new case of HIV infection occurred among adolescent girls or young women every two minutes. In the given situation, COVID-19 pandemic jeopardized the treatment and prevention services for HIV infection.^3^ According to WHO, 73 countries reported antiretroviral therapy (ART) disruptions during the pandemic in varying degrees.^4^ Although the lockdown was the need of the hour to break the transmission chain, it limited public mobility and access to various healthcare services. This study intends to address the evidenced threats faced by HIV care services during the pandemic; so that it helps in further research to combat such disruptions in care delivery during any public-health crisis.

###  Evidence before this study

 A substantial number of primary studies have been conducted representing different low and middle-income countries to understand the pandemic’s effect on HIV care services at the regional level. These studies proved that COVID-19 has impacted all aspects of the HIV healthcare system specifically treatment and testing services. To the best of our knowledge, studies that are objectively similar to our latest review curating shreds of evidence from March 2020 could be found. However, majority of the studies have focussed only on the adverse effects of the pandemic on HIV care delivery services, none has reported a robust framework for recommendations.

###  Added value of this study

 Despite of irreversible damage faced in the progress made in the fight against HIV; alternative, resilient approaches were adopted worldwide to combat the loss. In this review alongside assessing the aftermath of the pandemic on HIV care delivery services, we attempted to summarise what was successful in sustaining the services, innovative approaches that have evolved, and suggestions to accelerate the recovery.

 Therefore, this systematic review has been planned with a novel approach to achieve a deeper insight into addressing the compensatory measures which were adopted as temporary alternatives to alleviate the burden of interruptions alongside the negative outcomes of the pandemic on HIV care services along with reasonable recommendations for the healthcare advocates to envisage at the policy level.

###  Implications of all available evidence

 The available evidence will help in planning and in the implementation of the novel strategies which came up during the pandemic not only in HIV care delivery but also in routine healthcare programmes wherever applicable.

 This review attempted to address the reverberations of the coronavirus pandemic on HIV care service delivery in a multi-dimensional, holistic approach. It will guide to win over the losses as well as to protect the HIV care delivery services in any future public health crisis.

## Material and Methods

###  Protocol and registration

 A detailed plan for this systematic review was registered with the Open Science Framework (DOI: 10.17605/OSF.IO/74GHM). The Preferred Reporting Items for Systematic Reviews and Meta-Analyses (PRISMA) guidelines were followed for systematically reviewing the available evidences. Any deviation from the protocol has been described accordingly.

###  Strategy for searching and procedure for selection

####  Strategy for searching

 Search strategies were developed in collaboration with the research team. Databases namely PubMed, DOAJ, Science Direct were searched methodically to retrieve the eligible studies. The keywords “COVID-19”, “pandemic”, “HIV”, “HIV care continuum”, “impact”, “systematic review” were identified and then the key terms were customised to form the possible combination of search strings seeking to capture the most appropriate studies (Supplementary file 1) respectively for each database. The search strategy was restricted to papers in English language between December, 2019 to December,2022. Additionally, for other sources studies were searched in Google Scholar and using citation analysis of included studies from the three databases. NIH quality assessment tool and JBI Critical Appraisal Checklist (for qualitative studies) was used for assessing the quality of individual studies.

###  Inclusion and exclusion criteria

 The inclusion and exclusion criteria were set based on PICOT (Population, Intervention, Comparison, Outcome and Time) criteria.^5^ We included original research, short communications, report and viewpoint articles in English language, relevant to the repercussions of COVID-19 pandemic on services for HIV care. Studies without an available free full text, published in languages other than English, irrelevant to the scope of our study objective were excluded. Review articles, case studies, programme reports, policy documents, commentary and scientific letters were excluded.

###  Study selection

**Figure 1:**
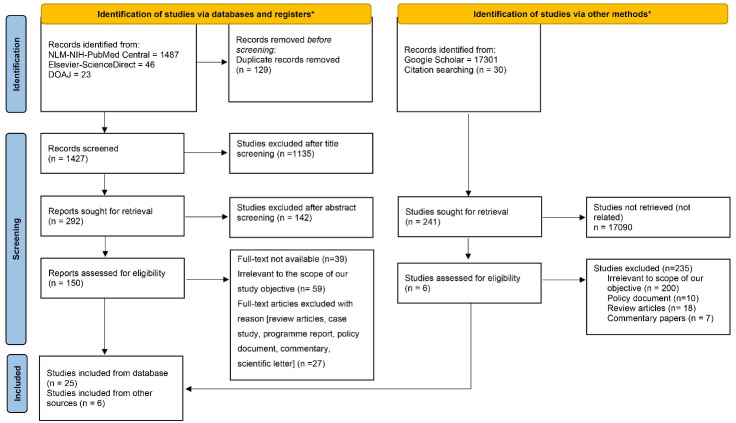
Preferred Reporting Items for Systematic Reviews and Meta-Analyses (PRISMA) flow diagram for selection of studies.

 Out of the 1556 records identified from the three electronic databases searching, 129 studies were found to be duplicate records. After removing the duplicate records, 1427 studies were title screened, out of which 292 were sought for retrieval. From here, 142 studies had to be excluded after abstract screening. After eligibility assessment, finally 25 studies met the methodological criteria and were found to be appropriate for the final inclusion in this review. Parallelly with regard to other sources, studies were retrieved from Google Scholar using customized search strings and some more studies were fetched using forward and backward citation analysis of the already selected studies (Figure 1). Finally, 31 studies were retained to be included in this review.

 The screening procedures were carried out independently by three authors (RC, DA and RT). Any disagreements among the authors were settled through discussion to reach a consensus, if needed. In scenarios where agreement could not be reached, disagreements were resolved by a senior author (MB).

###  Data collection

####  Data extraction and analysis

 Three authors (RC, DA, RT) independently extracted data from the included studies and any disagreement was resolved after discussing with a senior author (MB). We extracted data on study location, design, objective, target population, interventions or newer strategies adopted (if any) during pandemic period, primary and secondary outcomes using Microsoft Excel spreadsheet.

####  Quality assessment

**Figure 2:**
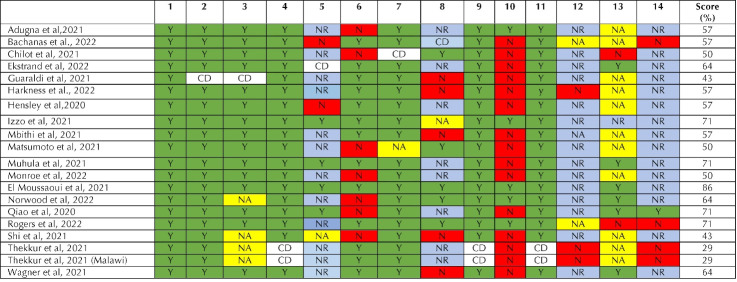
NIH Quality Assessment tool for observational cohort & cross-sectional studies. Yes (Y)- Green; No (N)-Red; Not Reported (NR)- Blue; Not Applicable (NA)- Yellow; Cannot Determine (CD)- White.

**Figure 3:**
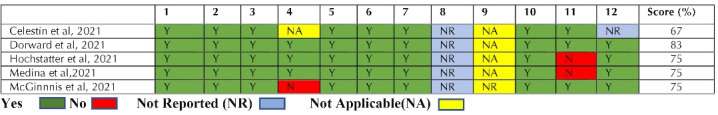
NIH Quality Assessment tool for Pre-Post studies without Control Group

**Figure 4:**
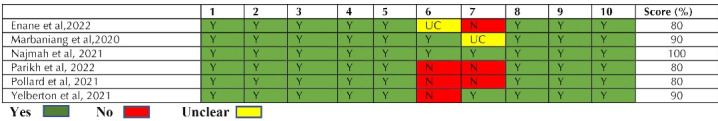
JBI Critical Appraisal Checklist for Qualitative Studies

 The quality of included studies were assessed using NIH quality assessment tools and JBI Critical Appraisal Checklist (for qualitative studies).^6^ Detailed coverage of the quality evaluation has been provided in Figures 2, 3 and 4. Three authors (RC, DA and RT) performed a collaborative quality assessment, and any differences were addressed by involving a senior author (MB). All 31 studies have been considered in this review. However, studies with fewer items checked in the quality assessment checklist (Supplementary file 1) received limited attention. The summary score of each study was calculated and expressed as percentage for both NIH and JBI quality assessment tools. The studies were categorized into four categories: poor (0–25%), fair (25–50%), good (50–75%) or excellent (75–100%).^7^

## Results

###  Characteristics of included studies

 The 31 included studies across the globe consists of high, low and middle-income countries namely USA, India, Kenya, Ethiopia, Italy, China, Haiti, South Africa, Netherlands, Vietnam, Guatemala, Belgium, Indonesia, Zimbabwe, Malawi and Uganda etc.

###  Findings

 We found, 39% of the included studies (12/31 studies) collected data from online repositories such as electronic health records of the healthcare facilities, available program data and 48% (15/31) studies used traditional offline mode of data collection from hospital settings and telephonic surveys. Qualitative research included (n = 6) in this review, primarily utilized methods such as in-depth interviews and focus-group discussion for gathering data.

 Based on the key highlights (Table 1), we have condensed the consequences of the pandemic on the HIV comprehensive care services under six major themes namely: (1) Lab services (2) Treatment and allied services, (3) Counselling services, (4) Outreach services, (5) Psycho-social impact, (6) Implementation of sustainable strategies.

**Table 1:** Summary of included studies

**Author and yea**r	**Study Objectives**	**Study Highlights**	
		**Strategies adopted**	**Service disruption & implication**
Adugna et al,2021^1^	The objective of this study was to assess the impact of COVID-19 pandemic on six aspects of HIV care services: VCT, Provider Initiated Counselling and Testing, antiretroviral therapy (ART), newly started ART, TB screening and lost to ART follow-up in Ethiopia.	Maintenance of HIV care services for PLHIVs already on ART Prescribing ART for multiple months with tailored service distribution.	Instances of attrition in HIV care offerings like VCT (voluntary counselling and testing) and PICT (provider-initiated counselling and testing services) Sub-optimal adherence of ART among newly diagnosed PLHIVs
Bachanas et al, 2022^8^	To study the treatment patterns prior to and amid the pandemic, and to evaluate the effectiveness of suitable interventions implemented via President’s Emergency Plan for AIDS Relief (PEPFAR).	Multi-month ART dispensing Facility based client centred services Community based client service adaptations Technological support for: Client tracking and tracing; virtual client support and education; communication and support Laboratory-services optimization Supply chain monitoring	Limited progress with delayed detection and treatment initiation in case of paediatric-population (CLHIV)
Celestin et al, 2021^9^	This study outlines alterations in the utilization, provision, and consistency of HIV services in Haiti during the 8 weeks preceding and following the diagnosis of the initial cases of COVID-19.	Use of MMD (Multi-month dispensing) and DAC (ART dispenses in community-based settings) Utilizing phone calls and SMS to actively engage in communication with people living with HIV (PLHIV)	Decreased HIV clinic visits. Interruption of ART refills both at clinic and community settings
Chilot et al, 2021^10^	To examine the immediate impact of COVID-19 on individuals with HIV attending ART clinics in Addis-Ababa of Ethiopia	Providing ART medication for 3-6 months	Missed ART appointment due to transport disruption Decreased earnings when going to a medical centre Limited availability of masks, sanitizers and non-medical assistance
Dorward et al, 2021^11^	To assess the effects of the lockdown on crucial aspects of HIV care specifically, HIV testing, the initiation of ART, and the continuation of care for HIV patients measured through ART collection visits and instances of absenteeism.	HIV services were generally maintained among people already receiving ART	Decrease in HIV testing and ART initiation
Ekstrand et al, 2022^12^	To study the novel measures of ART adherence and to analyse the reasons behind the anxiety faced by PLHIV in COVID pandemic	ART Medication for multiple months. Information about coronavirus transmission Solving individual barriers to clinic visits, prescription refills and simple techniques like deep breathing was provided via teletherapy	Fear of contacting COVID during clinic visits Stigma and discrimination regarding disclosure of their HIV status
Enane et al, 2022 ^13^	This research investigated how the coronavirus pandemic affected the provision of HIV services and engagement in care among adolescents in western Kenya.	Provision for youth peer mentor for sustainable HIV care Multi-month ART refills	Cuts in funding for HIV services Relocation of clinics specially designated for adolescents Shortage of health care workers due to strike
Guaraldi et al, 2021^14^	This study contrasted the 90–90–90 targets achieved during the COVID-19 pandemic in 2020 with the targets achieved from 2017 to 2019 in PLHIV	Same-day or rapid ART initiation programs and personalized interventions for vulnerable populations Availability of self-testing and rapid test screening outside of hospital environments	Vulnerable population were difficult to approach using telemedicine Target for HIV diagnosis, viral load suppression and provision of ART hindered during COVID
Harkness et al, 2022 ^15^	To record interruptions caused by coronavirus pandemic and the adaptive advancements in HIV services.	Telehealth services Providing remote service options like mailing testing kits and medicines. Flexible-community-based outreach methods	Disruption in: HIV testing services PrEP initiation Poor case management
Hensley et al, 2020^16^	This study aimed to understand the impact of restrictions due to the pandemic on the first pillars of the HIV care continuum in clinical settings.	Sensitive automated monitored tracking network for HIV ICs (HIV testing in risk-groups and Indicator conditions)	Drop in HIV testing rate Less patients were referred for new HIV diagnosis Advanced Stage of disease during entry in clinical care
Hochstatter et al, 2021^17^	This study compared substance use and HIV care before and during the pandemic using data collected weekly through an opioid relapse prevention and HIV management mobile-health Intervention(A-CHESS).	Introduction of mobile-health applications for retaining PLHIV and SUD (substance use disorder) in care during pandemic	Increased use of illicit substances. Missed ART medication Decreased confidence in maintaining-HIV appointments.
Izzo et al, 2021^18^	Evaluation of the effect of pandemic on HIV viral load and care continuum from March to November 2020.	Telemedicine and home delivery services for ART Counselling to maintain ART adherence.	Delay in ART refills, viral load and CD4 count Missed clinic visits due to fear of COVID infection
Marbaniang et al, 2020^19^	Evaluating the impact of anxiety on individuals with HIV in Pune, India, amidst the pandemic.	Sufficient stock of ART Use of short screening tools for monitoring anxiety levels among PLHIVs	Non-adherence to ART due to anxiety Financial and food insecurity Fear of infection
Mbithi et al, 2021^20^	This study determined the impact of the COVID-19 pandemic on HIV services through strengthened real-time surveillance	Increase in ART referral HIV self-testing	Decrease in ART testing Strike among health care professionals due to lack of protective equipment.
Matsumoto et al, 2021^21^	This research assessed the occurrence of SARS-CoV-2 and the social and behavioural effects of COVID-19 on the HIV care continuum.	Uninterrupted ART refills. Social health insurance schemes for PLHIVs Increased practice of COVID protective behaviours	Increase in psychological stress and financial burden Increase in risky health behaviours.
Medina et al, 2021^22^	To evaluate the decrease in HIV testing and screening programs for opportunistic infection during the COVID-19 pandemic.	Multi-month dispensing of ART for PLHIVs Implementation--of telemedicine platforms	Decrease in HIV testing Deaths due to inadequate diagnosis of opportunistic infections Diversion—of--healthcare workers
McGinnis et al, 2021^23^	Evaluating changes in HIV health care delivery and frequency of alcohol and tobacco use screening and also comparing HIV healthcare delivery by race/ethnicity and gender during and prior to the pandemic.	ART coverage maintained throughout the pandemic Virtual clinic visits-mostly virtual. ART prescriptions are routinely renewed through mail services without the need for a previous appointment. The prevalence of alcohol and tobacco consumption was lower in the time period of the pandemic.	In comparison to men living with HIV, a smaller proportion of women undergoing HIV care had both adequate ART coverage and successfully suppressed viral loads. Reduced frequency of screening for substance use among PLHIV
Muhula et al, 2021^24^	This study determined the disruption of healthcare-seeking behaviours, PrEP uptake and HIV testing and treatment services of PLHIV during the onset of COVID pandemic.	Significant increase in PrEP uptake Increased viral suppression	Missed ART medication due to increased pressure of food insecurity Shortage of medicines Fear of COVID infection
Monroe et al, 2022^25^	This study investigated the importance of ensuring continuity of care for maintaining the momentum in achieving the 90-90-90 goals and also considered PLHIV as a high priority group for any kind of intervention.	Consistent access to ART Increased use of telehealth	Decreased use of preventive service/prophylactic measures Reduced HIV lab monitoring
El Moussaoui et al, 2021^26^	This research investigated the impact of the consecutive waves of the COVID-19 pandemic in 2020, along with the resultant lockdown measures, on the HIV care process. Furthermore, it aimed to put forth potential strategies for sustaining efficient HIV prevention and care practices.	Telehealth alternatives were created to guarantee medical care through virtual and remote means Automatized follow-up for co-morbidities and co-infection screening.	Decrease in new HIV diagnosis and out-patient visits to HIV clinics. Less number of screening for hepatitis C and syphilis but increased screening for Chlamydia and Gonorrhoea
Najmah et al, 2021^27^	This study qualitatively investigated how women living with HIV, as well as mothers without HIV, perceive stigma. Additionally, it examined the challenges that women living with HIV (WLHIV) encountered in accessing HIV care amidst the COVID-19 pandemic.	Supportive health systems and peer education for health workers working for WLHIV Sexual and reproductive health rights and gender equality, particularly for WLHIV in the health curriculum	Increased stigma as compared to the pre-pandemic phase. Difficulty in access to health care services, which is exacerbated by the COVID-19
Norwood et al, 2022^28^	Examined the influence of the pandemic on the HIV care continuum at a major urban HIV clinic by assessing factors such as new patient appointments, mental health consultations, follow-up visits, and viral load suppression.	Behavioural health consultant was available to connect via telemedicine for PLHIV Primary healthcare services for PLHIV facing mental distress due to pandemic.	Increased mental illness in PLHIVs Decreased access to HIV testing Delay in HIV diagnosis. Decrease in outpatient medical encounters
Parikh et al, 2022^29^	Investigated the effects of the pandemic on Sexual and Reproductive Health (SRH) services, as well as the extent of challenges encountered by individuals living with HIV (PLHIV) when trying to access treatment.	Healthcare coverage schemes for PLHIV Nutritional supplementation: door- to-door deliveries of ART and food ration. Access to ART and SRH services for PLHIV without restrictions on travel.	Delay in receiving medication, unavailability of tests Difficulty in accessing SRH services. Stigma due to queuing outside ART centres
Pollard et al, 2021^30^	Examined the effects of COVID-19 related disturbances and innovative strategies for delivering HIV services to vulnerable groups in low- and middle-income nations.	PLHIV stable on treatment, ART was dispensed for them for 30 days from any govt. facility MMD was approved for 3 months during the pandemic ART can be picked up from any public centre across the country through home or community-based delivery	Decreased access to HIV facility-based testing services. Barriers due to lockdown from visiting facilities for ART pick-up Financial burden among PLHIV for fulfilling basic needs
Qiao et al, 2020^31^	This study identified the difficulties in HIV services that were linked to patient care results amid the COVID-19 pandemic.	Strengthen the ability of HIV-related institutions to withstand challenges by consistently assessing and improving their organizational resilience.	Disruption in follow-up service Delay in ART refills Increase in opportunistic infections
Rogers et al, 2022^32^	To record pandemic-related alterations aimed at reducing interruptions in HIV care and treatment for PLHIV.	Provision for additional ART supplies Reduction of non-urgent clinic visits	Poor retention in care ART adherence Viral load suppression
Shi et al, 2021^33^	For understanding how COVID 19 related restrictive measures impacted HIV care systems using real -time case reporting system during the first three months of 2020 among newly diagnosed PLHIVs.	Initiated Government policies for PLHIVs Promoted HIV self-testing	Delay in ART initiation among newly diagnosed PLHIVs Decrease in HIV testing
Thekkur et al, 2021^34^	This research assessed how the COVID-19 pandemic affected the detection, diagnosis, and treatment outcomes of TB cases, as well as HIV ART, using enhanced real-time surveillance.	Human resource support was provided to support and sustain ART service and HIV testing.	Voluntary male medical circumcision services (VMMC) were stopped Stock outs of HIV test kits.
Thekkur et al, 2021^35^	To compare pre-COVID service delivery (HIV & TB) as compared to the COVID period.	ART referral was maintained during pandemic	Reduction in HIV testing in the initial stage of the pandemic.
Wagner et al, 2021^36^	Examined the impact of the pandemic on HIV care in Uganda by analysing electronic health records from the country's major HIV care providers, both pre and post lockdown.	ART adherence was maintained due to self-reported Medical Event Monitoring System (MEMS)	Reduction in ART adherence Increased food insecurity Unavailability of screening for opportunistic infections.
Yelverton et al, 2021^37^	Comprehending the utilization of telehealth for HIV care, encompassing both medical and non-medical services and additionally, seeking to pinpoint obstacles to delivering remote services and devising strategies to enhance HIV care via telehealth during the pandemic.	Education and training sessions were conducted to enable telehealth. Wi-Fi enabled smartphones were handed over to the clients for their ease in contacting the healthcare provider.	Challenges related to technology Understanding digital skills Experiences of both clients and providers Economic disadvantage of client population Problem with reimbursement

####  1. Lab services

 One of the most studied topic among the literatures included in this review is the access to the laboratory services under the HIV comprehensive care services during the pandemic. Despite of considerable heterogeneity of settings and services, 14 out of the 31 included studies (45%) reported decreased testing or reduced lab monitoring during the COVID-19 pandemic resulting in delay in diagnosis.^15,22,25,26,28,29,33,35^

####  2. Treatment and allied services

 The pandemic has significantly impacted the provision of HIV-related treatment and related services. This study has arrived to a pooled opinions on the consequences of the coronavirus pandemic on the HIV-related treatment and other allied services. The most common observation was regarding anti-retroviral therapy. Shortage of medicines,^24^ delay in ART initiation,^11,33^ delay in receiving medications or ART refills,^9,18,29,31^resulting in sub-optimal or decreased ART adherence^1,36^ were reported. Other focal points derived were decreased screening of opportunistic infections,^1,9,36^ disruption in follow up services,^1,31^ decreased allied services like Voluntary Male Medical Circumcision ^34^ and preventive services.^25^

####  3. Counselling services

 In most of the countries HIV care comes in a comprehensive package. Along with treatment and reduction of viral load, screening of opportunistic infections, laboratory services, it is also equally dependent on the counselling services for risk reduction, safe practices, ensuring patients’ adherence to the care process in an integrated manner. Among the 31 included studies, only 2 studies covered the aspect of counselling services for HIV care. Both the studies reported that counselling services were ensured for the high-risk group amid the worldwide lockdown caused by the pandemic.^18,28^

####  4. Outreach activities

 The principal purpose of outreach activities in HIV comprehensive care services is case finding so that they become aware of their sero-status, and may get enrolled in care and treatment services.^38^ The provision of outreach services was negatively impacted by the diversion and strikes of health care personnel,^11,20,22^ cuts in funding for HIV care services,^13^ and decreased access to HIV care resources.^10^ Home delivery of ART medications and nutritional supplements was one of the strategies used to enhance outreach efforts.^29^

####  5. Psycho-social impact

 Social stigma and discrimination are known to be already prevalent among the high-risk groups for contracting HIV. The COVID-19 added to it and made it worse. 12 out of the included 31 studies has highlighted the various psycho-social factors faced by the high-risk groups during the COVID-19 pandemic worldwide. Financial burden^10,12,19,21,30^and fear of COVID-19^12,18,19,24^ has been the most common psycho-social impact in our findings, followed by increased stigma,^12,30,31^ mental distress or psychological stress,^17,21,28^ barriers due to lockdown like food insecurity^19,36^ and transport disruption^10,30^ leading to missed ART appointment.

####  6. Implementation of sustainable strategies

 During the COVID-19 pandemic, though the global scenario was depicting interruptions in accessibility of HIV facility based-care, but it has also accelerated the growth of alternative options with increased flexibility. The decrease in traditional facility-based clinic appointments were compensated by virtual telemedicine platforms.^9,12,15,18,22,23,25,26,28,37^ Sustainable strategies like home delivery of ART medications,^15,30^multi-month dispensing of ART for stable PLHIVs,^13,22^ provision of HIV self-testing to know one’s sero-status^14,20,33^has been reported. A study conducted by Parikh et al in Indian setting, reported nutritional supplementation and door-to-door ration delivery along with ART.^29^

## Discussion

 Multiple waves of COVID-19 outbreak prompted a huge public health response on a global scale, making it an unparalleled public health emergency. The pandemic forced HIV clinics to alter their approach from efforts to link and keep patients in care reducing in-person clinical appointments for PLHIVs, which severely weakened the HIV healthcare delivery system.^39^ This review has tried to examine the pandemic’s effect on the various facets of HIV care and listed suitable recommendations (Table 2) that were adopted to alleviate the burden of interruptions caused by the novel coronavirus pandemic.

 To examine the noteworthy influence of the COVID-19 pandemic on HIV care services, this analysis of 31 studies from various nations discovered consistent proof of substantial declines in the usage of comprehensive HIV care services during the subsequent pandemic waves.

**Table 2:** Recommendations and scope of future research

**Themes**	**Recommendations**	**Future research**
Lab services	In case of shortage of trained lab-personnel, task-sharing in specimen collection and point-of-care testing with non-lab professionals can be adopted.	Studies comparing the diagnostic accuracy of modern point-of-care technologies (infant diagnosis and viral load testing) performed by non-laboratory employees and laboratory professionals.
Treatment and allied services	Reduce administrative paperwork for initiation of ART. Separate provision for stable patients to “Fast track services”. Real-time monitoring of ART adherence using “Pill count” strategies.^40^ Stable PLHIVs on ART can be offered more of virtual consultation and multi-month ART refills instead of frequent in-person visits.	Research using pathway-review techniques to reduce unnecessary tasks. Cost-Benefit analysis for assessing ART adherence. Evidence on outcome associated with less frequent clinical visits or ART refills beyond 6 months for various populations.
Counselling services	Counselling techniques that include motivational interviewing and cognitive behavioural therapy Digital interventions using virtual platform for face-to-face interaction Mobile teams	Research protocols using Behaviour Change theories.
Outreach services	Making ART medications available at fixed locations outside health facility such as “external pick-up points” or “community drug distribution points”.^40^	Client preferences regarding collection of ART on the basis of age, population and setting
Psychosocial impact	Peer-driven and family-based intervention to promote mental health and prevent negative behaviour. For example, youth models like teen clubs in Malawi and Scholar model in Zambia.^41^	Specifically designed interventions for PLHIVs (adolescents and young adults) with disabilities, mental health conditions, acquired HIV perinatally and those with extreme poverty.
Implementation of sustainable strategies	Integration of services to reduce missed opportunities or drop-outs and to enhance ART adherence support, for example appointment reminders, incentives to attend post ART initiation visits etc. Extra clinic hours on evenings and weekends, family models (WHO recommended) can be adopted. People-centred care and specific models for nonstable patients.	Integration of HIV care with other routine health services to generate evidence on out-of-pocket health expenditure, effect of incentive on adherence etc.

**Figure 5:**
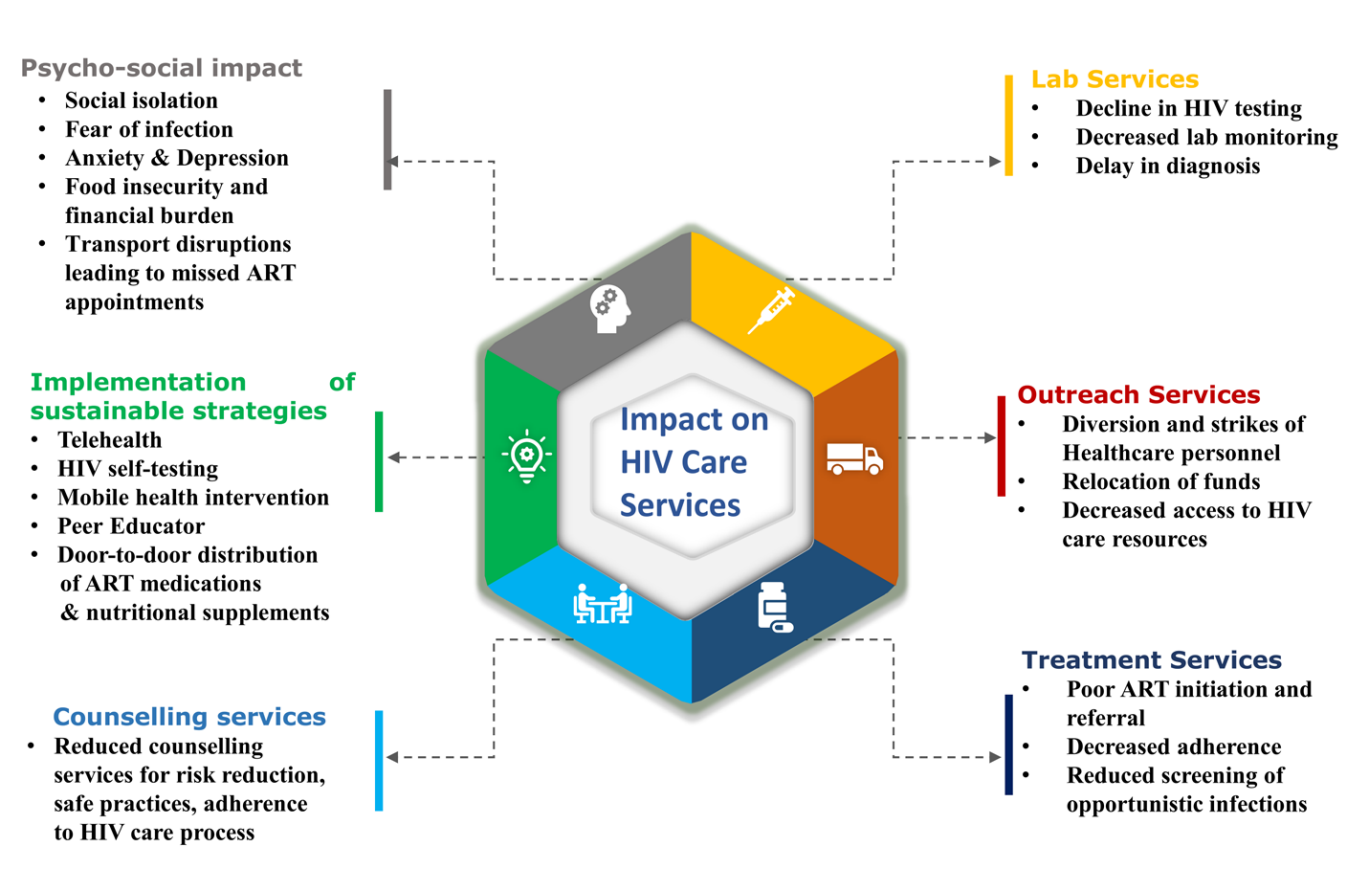
Framework showing impact of COVID-19 on the HIV Care cascade

 Although a meta-analysis was not possible due to a lack of quantitative summary from the available literature, this review has tried to summarize the repercussions of the coronavirus pandemic on HIV comprehensive care services under six major themes and has been discussed using a framework (Figure 5). Results revealed that the majority of the studies reported decreased testing or reduced lab monitoring,^15,22,25,26,28,29,33,35^ ultimately leading to a delay in diagnosis and hence interrupting the treatment cascade.^9,11,18,29,31,33^

 It has been found that few countries have adopted multi-month dispensing of drugs ^8,13,22,32^while most of the countries could not, due to a shortage of drugs. Such a buffer to health systems against external shocks is advisable. An interrupted supply of drugs under any unforeseen situations will hinder the progress of removing HIV/AIDS as a public health threat by 2030. Moving forward, countries should maintain a steady supply of ART drugs, especially keeping in mind of such public health crisis.

 HIV comprehensive care components are highly interdependent. One such component is the counselling services. From the findings, it can be inferred that ensuring the counselling service was compromised during the COVID-19 pandemic.

 This review has not only highlighted the detrimental points that the world has seen regarding HIV care services during the pandemic but also highlighted the potential alternatives or compensatory measures that have been adopted to bypass the threat. Despite the diversion and strikes of health care personnel,^11,20,22^ cuts in funding for HIV care services^13^ and decreased access to care resources,^10^ our findings revealed some best possible alternatives like door-to-door distribution of ART medications and nutritional supplements that were adopted to combat the issue.^29^

 As HIV mostly deals with a marginalized section, there is already a stigma and discrimination prevalent amongst the population. COVID-19 being another infectious infection, added fear,^12,18,19,24^ psychological stress,^17,21,28^ barriers due to lockdown like transport disruption,^10,30^ food,^19,36^and financial insecurity^10,19,21,30^ to it and magnified the condition.

 In high-resource settings, interruptions in the accessibility of HIV comprehensive care services were compensated by the accelerated provision of alternative options with increased flexibility. For example, teleconsultation or virtual platforms,^9,12,18,22,23,25,26,28,37^ was adopted for the smooth functioning of routine HIV care services. In some countries, home delivery^30^ of ART medications, multi-month dispensing of ART,^6,8,13,32^ provision of self-testing^14,20,33^ and nutritional support^29^ were adopted.

 For improving service provision “Differentiated service delivery” (DSD) model, a people-centric approach to HIV service delivery have been successfully implemented in countries like Malawi, South Africa, and Zambia.^42^ This kind of model can change the “when, where, who, and what” of HIV service delivery to a diverse group of PLHIVs while maintaining the principles of public health approach.

## Strengths & Limitations

 This study has incorporated the most recent data from primary studies since the inception of the COVID-19 pandemic till December 2022, which covers the peak of the pandemic in most countries. Secondly, it has consolidated the consequences of the pandemic and related changes on the utilization of HIV care services. Thirdly, the risk of bias in the selection of studies has been minimized using standard quality assessment tools. Citations from retrieved publications were checked and necessary studies have been included. A clear explanation of the PRISMA flow diagram, formulation of search strategy, data curation, and analysis methods to eliminate any potential confusion has been provided. The results have been interpreted with a special focus on the pandemic scenario, hence broad generalization of inference may be restricted to situations related to any public health crisis only. Also, some potential primary studies with significant results might have been missed out due to limited access to multiple databases. Excluding literature in non-English language is a limitation of this study. Meta-analysis could not be done due to the lack of a quantitative summary of available literature. The effectiveness of all the compensatory measures that has been addressed from various literatures may not be uniform and has the potential to vary across different settings, here leaves scope for future research.

## Recommendations

 Same-day start of ART or rapid ART initiation, provision of ART initiation outside-the-clinic, reducing the frequency of clinical visits for stable patients via virtual platforms, and provision for ART refills from external pick-up points or community drug distribution points^40^ will help in fast-tracking HIV care services. This study recommends measuring adherence using novel strategies like ‘pill count’^40^; tracing and re-engagement of drop-out cases in care; psychosocial support for people living with HIV; task sharing for diagnostic services and integration of HIV service deliveries. These recommendations may help the programme managers and policy-makers in developing countries to design models and strategies that will help in the smooth functioning of the HIV care continuum and the standard of care for PLHIVs to mitigate any further public health crisis.

## Conclusion

 This study has tried to consolidate the evidence available from a global perspective. Though the COVID-19 containment measures like quarantine and lockdown measures have put the HIV care continuum in many parts of the world into a challenging situation, the transition with new innovative, sustainable care paradigms, and resilient interventions calibrated to combat such vulnerabilities are vital to ensure continuity of care and may prove to be the building blocks in removing HIV-AIDS as a public health threat. Strong organizational preparedness, inter-sectoral coordination, enhanced and improved service delivery techniques, need based timely and effective support to the community are required to combat this public health crisis.

## Acknowledgements

 The authors express their heartfelt gratitude to the staffs at the ICMR-National Institute of Cholera and Enteric Diseases in Kolkata, India, for their assistance and backing.

## Competing Interests

 The authors state that there are no possible conflicts of interest regarding the research, authorship and/or publication of this article.

## Data Availability Statement

 All data produced or examined for this article are incorporated within it. Further explanations can be provided by the corresponding author upon request.

## Ethical Approval

 Already published data in various literatures have been used, so ethics approval and consent were not required for this study.

## Supplementary Files


Supplementary file 1 contains the NIH and JBI quality assessment checklists.
