# Supplementary material for: Syndemic effect of COVID-19 outbreak on HIV care delivery around the globe: A systematic review using narrative synthesis
Source: Health Promot Perspect. 2023 Dec 16;13(4):243–53. doi: 10.34172/hpp.2023.30 (PMC10790127; doi:10.34172/hpp.2023.30)
Supplement: Supplementary file 1 — contains the NIH and JBI quality assessment checklists. [file hpp-13-243-s001.pdf]

Chakrabarti et al, **Health Promotion Perspectives,** 2023, 13(4), 243-S1.
doi: 10.34172/hpp.2023.30
https://hpp.tbzmed.ac.ir

I. Database search strings for PubMed

Search	Query	Results
#7	Search: **#1 AND #2 AND #3 AND #4** Filters: **Free full text, from 2019/12 - 2022/12**	1,487
#6	Search: **#1 AND #2 AND #3 AND #4** Filters: **from 2019/12 - 2022/12**	1,776
#5	Search: **#1 AND #2 AND #3 AND #4**	1,810
#4	Search: **(HIV care) OR (HIV treatment) OR (HIV service delivery)**	256,508
#3	Search: **(HIV) OR (HIV-1) OR (human immunodeficiency virus)**	420,155
#2	Search: **(covid19) OR (SARSCOV2) OR (corona virus)**	321,458
#1	Search: **(impact) OR (effect)**	11,625,433

I. The National Institutes of Health (NIH) quality assessment tool for observational cohort and cross-sectional studies<br>**Website:** https://www.nhlbi.nih.gov/health-topics/study-quality-assessment-tools			
Major Components	Response options		
1. Was the research question or objective in this paper clearly stated?	Yes	No	Cannot Determine/ Not Applicable/ Not Reported
2. Was the study population clearly specified and defined?	Yes	No	Cannot Determine/ Not Applicable/ Not Reported
3. Was the participation rate of eligible persons at least 50%?	Yes	No	Cannot Determine/ Not Applicable/ Not Reported
4. Were all the subjects selected or recruited from the same or similar populations (including the same time period)? Were inclusion and exclusion criteria for being in the study prespecified and applied uniformly to all participants?	Yes	No	Cannot Determine/ Not Applicable/ Not Reported
5. Was a sample size justification, power description, or variance and effect estimates provided?	Yes	No	Cannot Determine/ Not Applicable/ Not Reported
6. For the analyses in this paper, were the exposure(s) of interest measured prior to the outcome(s) being measured?	Yes	No	Cannot Determine/ Not Applicable/ Not Reported
7. Was the timeframe sufficient so that one could reasonably expect to see an association between exposure and outcome if it existed?	Yes	No	Cannot Determine/ Not Applicable/ Not Reported
8. For exposures that can vary in amount or level, did the study examine different levels of the exposure as related to the outcome (e.g., categories of exposure, or exposure measured as continuous variable)?	Yes	No	Cannot Determine/ Not Applicable/ Not Reported
9. Were the exposure measures (independent variables) clearly defined, valid, reliable, and implemented consistently across all study participants?	Yes	No	Cannot Determine/ Not Applicable/ Not Reported
10. Was the exposure(s) assessed more than once over time?	Yes	No	Cannot Determine/ Not Applicable/ Not Reported
11. Were the outcome measures (dependent variables) clearly defined, valid, reliable, and implemented consistently across all study participants?	Yes	No	Cannot Determine/ Not Applicable/ Not Reported
12. Were the outcome assessors blinded to the exposure status of participants?	Yes	No	Cannot Determine/ Not Applicable/ Not Reported
13. Was loss to follow-up after baseline 20% or less?	Yes	No	Cannot Determine/ Not Applicable/ Not Reported
14. Were key potential confounding variables measured and adjusted statistically for their impact on the relationship between exposure(s) and outcome(s)?	Yes	No	Cannot Determine/ Not Applicable/ Not Reported
Quality Rating	Good	Fair	Poor

Additional Comments (If Poor, please state why):			
**II. The National Institutes of Health (NIH) quality assessment tool for before-after (Pre-Post) study with no control group**<br>**Website:** https://www.nhlbi.nih.gov/health-topics/study-quality-assessment-tools			
Major Components	Response options		
1. Was the study question or objective clearly stated?	Yes	No	Cannot Determine/ Not Applicable/ Not Reported
2. Were eligibility/selection criteria for the study population prespecified and clearly described?	Yes	No	Cannot Determine/ Not Applicable/ Not Reported
3. Were the participants in the study representative of those who would be eligible for the test/service/intervention in the general or clinical population of interest?	Yes	No	Cannot Determine/ Not Applicable/ Not Reported
4. Were all eligible participants that met the prespecified entry criteria enrolled?	Yes	No	Cannot Determine/ Not Applicable/ Not Reported
5. Was the sample size sufficiently large to provide confidence in the findings?	Yes	No	Cannot Determine/ Not Applicable/ Not Reported
6. Was the test/service/intervention clearly described and delivered consistently across the study population?	Yes	No	Cannot Determine/ Not Applicable/ Not Reported
7. Were the outcome measures prespecified, clearly defined, valid, reliable, and assessed consistently across all study participants?	Yes	No	Cannot Determine/ Not Applicable/ Not Reported
8. Were the people assessing the outcomes blinded to the participants' exposures/interventions?	Yes	No	Cannot Determine/ Not Applicable/ Not Reported
9. Was the loss to follow-up after baseline 20% or less? Were those lost to follow-up accounted for in the analysis?	Yes	No	Cannot Determine/ Not Applicable/ Not Reported
10. Did the statistical methods examine changes in outcome measures from before to after the intervention? Were statistical tests done that provided p values for the pre-to-post changes?	Yes	No	Cannot Determine/ Not Applicable/ Not Reported
11. Were outcome measures of interest taken multiple times before the intervention and multiple times after the intervention (i.e., did they use an interrupted time-series design)?	Yes	No	Cannot Determine/ Not Applicable/ Not Reported
12. If the intervention was conducted at a group level (e.g., a whole hospital, a community, etc.) did the statistical analysis take into account the use of individual-level data to determine effects at the group level?	Yes	No	Cannot Determine/ Not Applicable/ Not Reported
**Quality Rating**	Good	Fair	Poor
Additional Comments (If Poor, please state why):			

JBI CRITICAL APPRAISAL CHECKLIST FOR QUALITATIVE RESEARCH

Reviewer_____ ____

Date_________________________________

Author____ Year_________ Record Number_________

	Yes	No	Unclear	Not applicable
1. Is there congruity between the stated philosophical perspective and the research methodology?	☐	☐	☐	☐
2. Is there congruity between the research methodology and the research question or objectives?	☐	☐	☐	☐
3. Is there congruity between the research methodology and the methods used to collect data?	☐	☐	☐	☐
4. Is there congruity between the research methodology and the representation and analysis of data?	☐	☐	☐	☐
5. Is there congruity between the research methodology and the interpretation of results?	☐	☐	☐	☐
6. Is there a statement locating the researcher culturally or theoretically?	☐	☐	☐	☐
7. Is the influence of the researcher on the research, and vice- versa, addressed?	☐	☐	☐	☐
8. Are participants, and their voices, adequately represented?	☐	☐	☐	☐
9. Is the research ethical according to current criteria or, for recent studies, and is there evidence of ethical approval by an appropriate body?	☐	☐	☐	☐
10. Do the conclusions drawn in the research report flow from the analysis, or interpretation, of the data?	☐	☐	☐	☐

Overall appraisal: Include ☐ Exclude ☐ Seek further info ☐

Comments (Including reason for exclusion)

____

____

____
